# Application and research progress of single cell sequencing technology in leukemia

**DOI:** 10.3389/fonc.2024.1389468

**Published:** 2024-08-29

**Authors:** Dan Xie, Bangquan An, Mingyue Yang, Lei Wang, Min Guo, Heng Luo, Shengwen Huang, Fa Sun

**Affiliations:** ^1^ Medical College, Guizhou University, Guiyang, China; ^2^ Guizhou Provincial People’s Hospital, Guiyang, Guizhou, China; ^3^ State Key Laboratory of Functions and Applications of Medicinal Plants, Guizhou Medical University, Guiyang, Guizhou, China; ^4^ Guizhou Provincial Engineering Research Center for Natural Drugs, Guiyang, Guizhou, China

**Keywords:** leukemia, single-cell sequencing, acute myeloid leukemia, heterogeneity, leukemia cells

## Abstract

Leukemia is a malignant tumor with high heterogeneity and a complex evolutionary process. It is difficult to resolve the heterogeneity and clonal evolution of leukemia cells by applying traditional bulk sequencing techniques, thus preventing a deep understanding of the mechanisms of leukemia development and the identification of potential therapeutic targets. However, with the development and application of single-cell sequencing technology, it is now possible to investigate the gene expression profile, mutations, and epigenetic features of leukemia at the single-cell level, thus providing a new perspective for leukemia research. In this article, we review the recent applications and advances of single-cell sequencing technology in leukemia research, discuss its potential for enhancing our understanding of the mechanisms of leukemia development, discovering therapeutic targets and personalized treatment, and provide reference guidelines for the significance of this technology in clinical research.

## 1 Introduction

**Figure 1:**
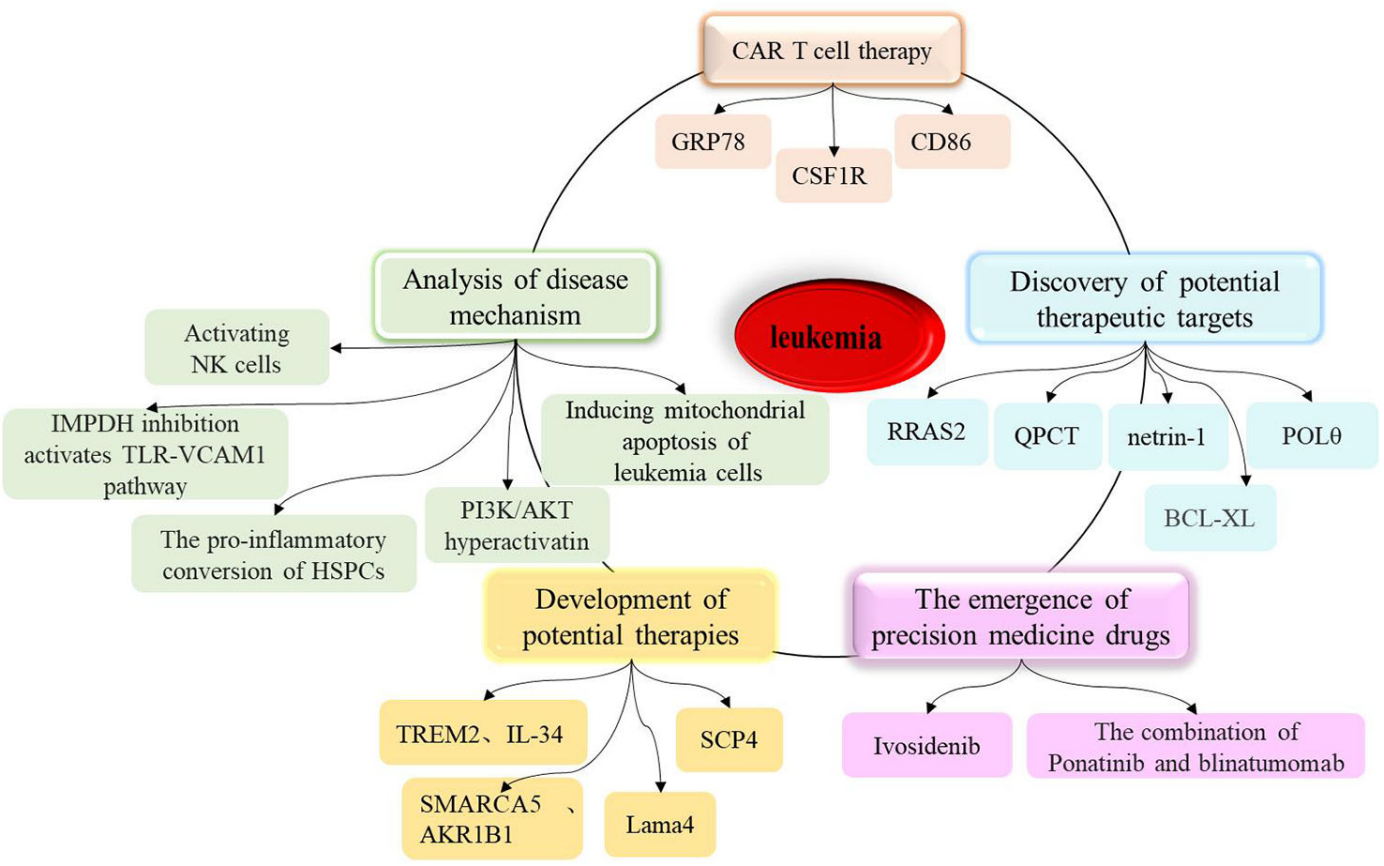
A summary of research progress on leukemia, including the analysis of disease mechanisms, the development of potential therapies, the discovery of new therapeutic targets, the marketing of precision therapeutic drugs, and the sustained remission of leukemia by CAR T cells.

Leukemia is a malignant blood disease caused by the abnormal proliferation and uncontrolled differentiation of hematopoietic cells. Over recent years, significant progress has been made in leukemia research, including the elucidation of disease mechanisms, the development of potential therapies, the discovery of new treatment targets, the emergence of precision treatment drugs, and the sustained remission of leukemia with chimeric antigen receptor (CAR)-T cells (as shown in **Figure 1**). In addition, scientists have identified a previously unknown mechanism, demonstrating that the direct AMPK activator GSK621 can activate AMPK/PERK to induce mitochondrial apoptosis in leukemia cells. Furthermore, in xenograft animal models, the combination of GSK621 and venetoclax enhanced the anti-leukemia activity of venetoclax (1). Furthermore, the inhibition of IMPDH can induce overactivation of Toll-like receptor (TLR)-TRAF6-NF-κB signaling and upregulate the adhesion molecule VCAM1, thus contributing to the anti-leukemia effects of IMPDH inhibitors (2).

In chronic lymphocytic leukemia (CLL), research by Ecker V. et al. shows that CLL cells critically depend on mechanisms to fine-tune PI3K/AKT activity, allowing sustained proliferation and survival, while avoiding ROS-induced cell death (3). Wang Y. et al. found that venetoclax directly activates Natural killer (NK) cells, enhancing their cytotoxicity against acute myeloid leukemia (AML) both *in vitro* and *in vivo*, and venetoclax promotes mitochondrial respiration and ATP synthesis via the NF-κB pathway, thereby facilitating immunological synapse (IS) formation in NK cells (4). Other studies have identified and characterized how the chromatin remodeling complex SMARCA5 mediates the abnormal chromatin accessibility in acute myeloid leukemia (AML) and revealed the important role of AKR1B1 in leukemia development (5). From the perspective of lipid metabolism, Guo HZ et al. explored new mechanisms of immune escape and therapeutic resistance in leukemia, providing new directions for subsequent clinical treatments (6). To develop more effective cancer therapies, scientists from the Dana-Farber Cancer Institute and their collaborators revealed the targetable dependency of acute leukemia patients on the PI3Kγ-PAK1 signaling pathway (7). Additionally, in the microenvironment of acute myeloid leukemia, healthy hematopoietic stem cells and progenitor cells (HSPC) promote the occurrence of inflammation (8). Furthermore, a study developed biomimetic vesicles by infusing HSPC membrane with liposomes (HSPC liposomes), and experiments validated that the vesicles carrying chemotherapy drug cytarabine (Ara-C@HSPC-Lipo) can significantly inhibit the proliferation of leukemia cells, induce apoptosis and differentiation of leukemia cells, and reduce the number of leukemia stem cells, providing a pathway for leukemia treatment (9).

The elucidation of these mechanisms may hold significant promise for the treatment of leukemia. Moreover, the development of CAR-T cells targeting GRP78 has led to the successful targeting and destruction of AML cells (10). In 2022, researchers used domain-focused CRISPR screening and discovered that AML cells may rely on a previously little-known protein called SCP4 for survival; further research is expected to develop new targeted therapies for AML (11). It is worth noting that the discovery of many potential therapeutic targets such as netrin-1, QPCT, POLθ, and RRAS2 has laid the theoretical and experimental foundation for subsequent clinical research (12–18).On the other hand, in recent decades, materials technology has also demonstrated significant advantages in enhancing current leukemia therapies and has garnered increasing attention (19).

**Figure 2:**
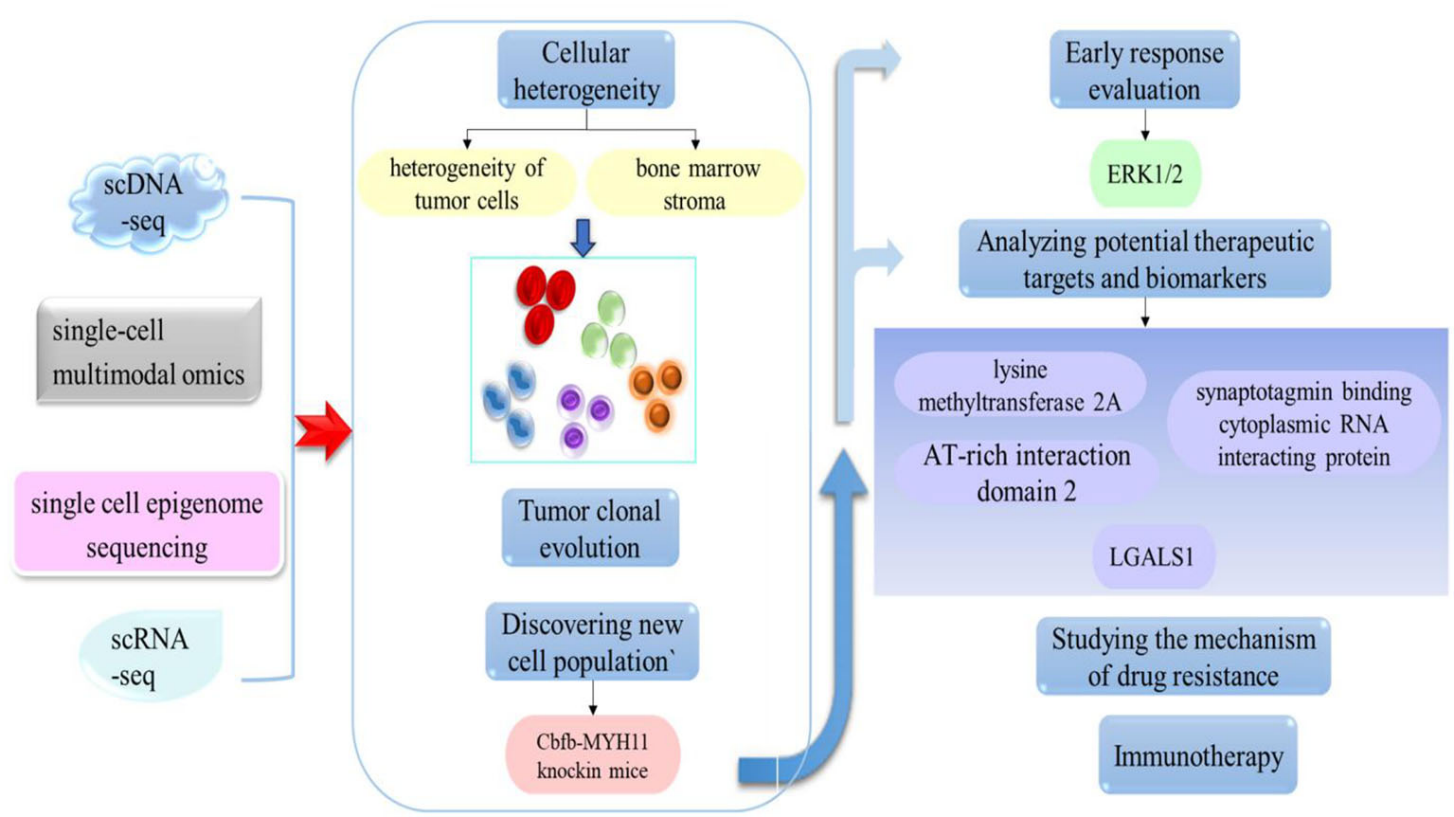
The application of single-cell technology in leukemia. By applying this approach, it is possible to investigate cell heterogeneity, the clonal evolution of tumors, discover new cell populations, and analyze potential therapeutic targets.

The research results summarized above indicate that significant progress has been made in the study of leukemia over recent years, providing strong scientific evidence for further research and offering important insights for the development of more effective treatment strategies and drugs. However, the high heterogeneity and complexity of this disease remain a significant challenge for researchers and clinicians. Traditional research methods often only provide information at the population level and cannot capture differences and key changes in individual cells. With the continuous advancement of technology, single-cell sequencing has become an important tool for studying cell biology in relation to medical status, thus providing a new approach for us to gain a deeper understanding of cell diversity and function. By applying this technology, we can further understand the mechanisms, molecular characteristics, and cellular heterogeneity of leukemia, and provide important guidance and breakthroughs for the diagnosis and treatment of leukemia (as shown in **Figure 2**). Therefore, in this article, we review the recent development of single-cell sequencing technology and its application in leukemia research, and introduce the latest research progress, focusing on the application of single-cell sequencing technology for the analysis of leukemia heterogeneity, chemotherapy resistance, immunotherapy, the discovery of potential therapeutic targets, and the clonal evolution of tumors. By comprehensively analyzing these aspects, we explore the potential and challenges of single-cell sequencing technology in leukemia research, as well as its prospects for clinical application in the treatment of leukemia.

## 2 Overview of single-cell sequencing technology

Traditional cytological research is typically based on mean data from a large number of cells, while single-cell sequencing technology can resolve and identify the heterogeneity of individual cells, thus providing more detailed and accurate information. The main steps of sequencing include single-cell isolation, cell lysis, gene amplification, library construction, sequencing, and data analysis. Single-cell isolation methods include mechanical separation, flow cytometry, and microfluidic chips. After sequencing, the sequencing data needs to be analyzed and interpreted. This crucial data analysis step includes quality control, alignment, gene expression quantification, differential expression analysis, clustering analysis, and functional annotation. The purpose of data analysis is to extract useful information from a large body of sequencing data, thereby revealing the specific characteristics and functions of cells.

Scientists first developed single-cell RNA sequencing in 2009 (20); since then, and with the optimization of high-throughput sequencing technology, researchers have continuously improved and developed new methods to improve the accuracy, throughput and resolution of sequencing. During the same period, several commercial companies launched specialized single-cell sequencing platforms, including 10x Genomics and Drop-seq. In addition to single-cell RNA sequencing, other technologies such as single-cell DNA sequencing, single-cell proteomics, and single-cell epigenetics have emerged, thus promoting the rapid development of single-cell multi-omics technologies. As a high-resolution genomic technology, single-cell sequencing has been widely applied in many fields, including cancer research and immunology.

**Figure 3:**
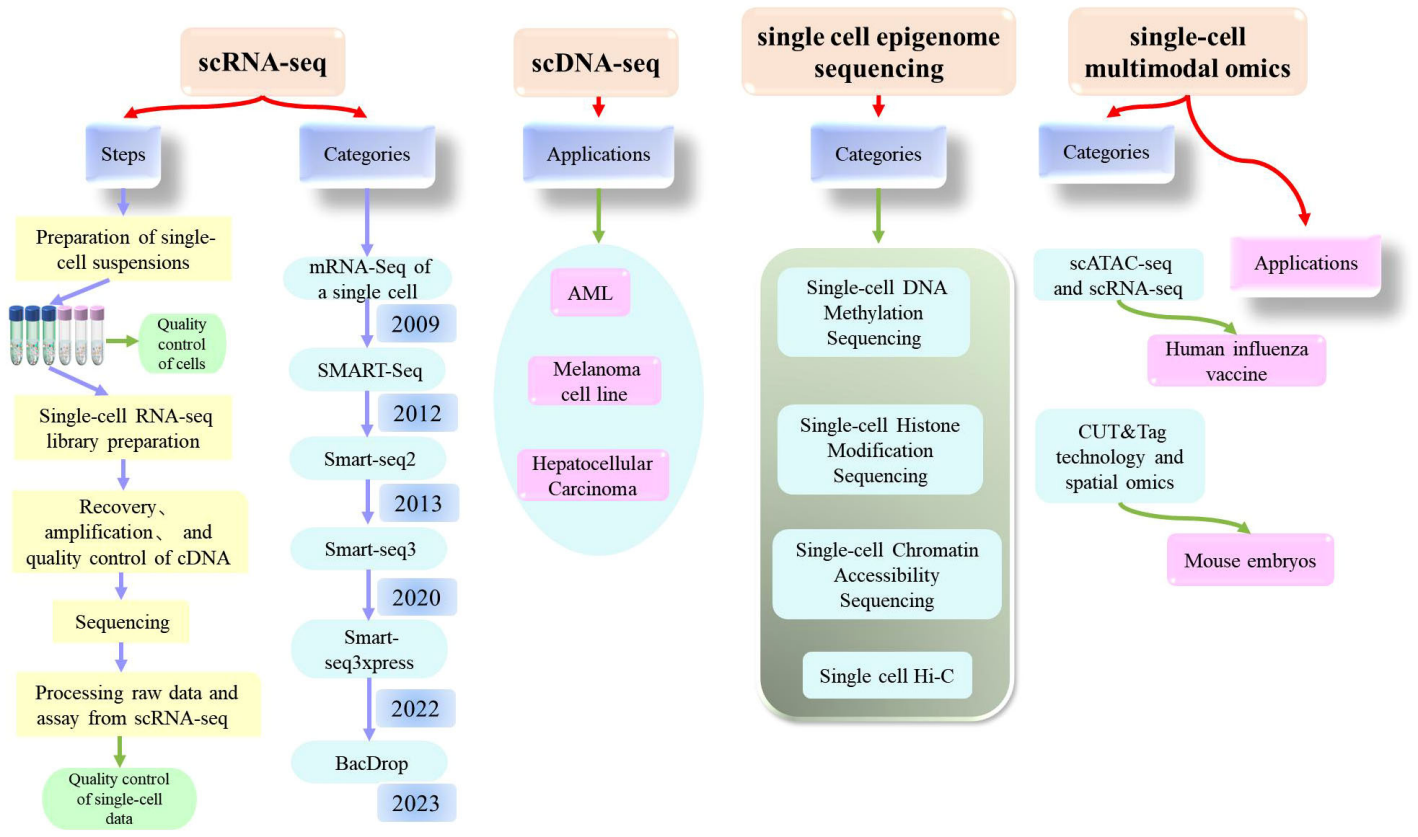
Comprehensive explanation of single cell sequencing (scRNA-seq, scDNA-seq, single cell epigenome sequencing, single-cell multimodal omics) which includes the steps, categories, applications.

Today, single-cell sequencing technology is a powerful tool that can provide significant insights into the heterogeneity and functionality of cells, offering new concepts and methods for the diagnosis and treatment of diseases. It is expected that single-cell technology will further drive the development and application of life sciences in the future. Here, we introduce the most common types of single-cell sequencing technologies (**Figure 3**).

### 2.1 Single cell RNA sequencing

Single-cell RNA sequencing (scRNA-seq) is a high-throughput sequencing technique that can determine RNA transcripts at the level of individual cells. This technique can provide significant information about the cellular transcriptome, including mRNA, rRNA, tRNA, and non-coding RNA. Single-cell RNA sequencing was first published by Tang et al. in 2009 (20). Since then, scRNA-Seq has been widely used in various fields (21–26). As biotechnology continues to evolve, researchers have also experimented with technological innovations and have established a wide range of sequencing technologies, including STRT-Seq, Smart Seq, CEL-Seq, Fluidigm C1, Smart-SeQ2, MARS-Seq, Cyto-Seq, Drop-Seq, inDrop-Seq, and VASA-seq (27).

Of these, the single-cell RNA sequencing method based on plates with complete transcript coverage (the SMART-Seq method) is very useful for identifying candidate biomarkers, SNPs, and mutations, and represents a milestone technology that was first published by the Ramsköld team in 2012 (28). A year later, the Picelli team introduced Smart-seq2 technology, which improved detection, coverage, bias, and accuracy compared to the Smart-seq, and also reduced costs (29, 30). Building on this, the Rickard team developed Smart-seq3 technology, which was published in 2020 and represents a low-cost solution for single-cell full-length transcriptomes (31). Compared to Smart-seq2, Smart-seq3 increases the length of transcripts and further improves sensitivity, reducing library construction costs by more than ten-fold. However, this method was still not able to achieve the high throughput detection of genes with reduced cost; thus, the same team subsequently developed Smart-seq3xpress (32). This technology further reduced sequencing costs compared to Smart-seq3 and produced high levels of data quality with a higher gene detection rate; this technique met the requirements for efficient and detailed cell clustering and was suitable for large-scale cell atlas construction.

In addition to the clear improvements made to these technologies, significant progress has also been made in the single-cell transcriptome sequencing of microorganisms. Researchers have recently developed BacDrop, a technique that is highly compatible with the number of cells in the experiment; The authors of this study suggested that scRNA-seq technology may hold significant promise for the study of microorganisms (33).

### 2.2 Single cell DNA sequencing

Single-cell DNA sequencing technology is a method for analyzing genomic DNA from individual cells. The principle of this technique is to amplify the minimal whole-genome DNA from isolated single cells, obtain high coverage of the complete genome, and perform high-throughput sequencing. Many tools and methods have been developed and used for single-cell DNA sequencing technology, including SCSilicon, SimSCSnTree, SCOPE, SECEDO, doubletD, ProSolo, Phylvar, Linked-read analysis, and SIEVE (34–42). Recently, scientists have developed a technology referred to as CODEC to improve the accuracy of next-generation sequencing technology and is expected to detect rare cancer mutations in blood samples and diagnose latent mutations in rare diseases at relatively low cost (43). These analytical methods not only detect doublets in scDNA-seq data, but also effectively cluster tumor cells to obtain more accurate results.

Although single-cell methods for the analysis of RNA and proteins have significantly extended our understanding of cellular diversity, many fundamental questions in biology and important medical applications require the analysis of DNA in single cells (44). As with scRNA-seq, scDNA-seq technology can be applied for many different applications in many fields. In 2018, researchers used single-cell analysis to investigate patients with AML; this research successfully revealed the clonal evolution of different patients. Other researchers used scDNA-seq to reveal the complex mechanisms underlying resistance to quizartinib in patients with AML (45, 46). In addition, Guo et al. used scDNA-seq to investigate the genomic characteristics of liver cancer at the single-cell level, and discovered a new prognostic marker, CAD, that could provide key information relating to the clinical diagnosis, prognosis, and treatment of liver cancer (47). Velazquez-Villarreal et al. performed shallow single-cell sequencing of the genomic DNA of 1475 cells from the COLO829 cell line by using an emerging droplet-based shallow genome sequencing technology; this research allowed the investigation of cancer with far higher resolution (48). These findings indicate that scDNA-seq is expected to have a significant biomedical and clinical impact in the future.

### 2.3 Single cell epigenome sequencing

Since different cell types with different epigenetic characteristics are always mixed together in tissues or organs, single-cell analysis can provide a universal solution for dissecting their inherent complexity (49). This technique is referred to as single-cell epigenetic sequencing, a method used to investigate epigenetic modifications in individual cells. Epigenetics refers to the phenomenon of regulating gene expression by specific mechanisms, including DNA methylation, histone modification, chromatin remodeling and three-dimensional spatial conformation. Single-cell transcriptome sequencing helps to decode cell phenotypes, while single-cell epigenetic sequencing technology helps further decode cell heterogeneity at the epigenetic level and analyze the fine regulatory mechanisms of epigenetics at the molecular level, so as to achieve the purpose of molecular decoding (50). In the following section, we describe four of the most commonly used single-cell epigenetic sequencing techniques.

#### 2.3.1 Single-cell DNA methylation sequencing

This technique can determine the DNA methylation pattern of a single cell. DNA methylation is a common epigenetic modification that plays a role in regulating both gene transcription and expression. In 2021, Liu et al. used single-cell methylation sequencing technology (snmC-seq2) to systematically analyze 45 brain regions in adult mouse brains and identified a total of 103,982 single-cell methylomics data, including 95,815 neurons and 8,167 other brain cells (51). Professor Chang Lu’s team conducted a study related to the high-throughput analysis of single-cell DNA methylomics, which described the application of a droplet-based microfluidic technology (Drop-BS) that could reveal the heterogeneity of cell types (52). Drop-BS offers a promising solution for single-cell methylomics studies that require the detection of large cell populations.

#### 2.3.2 Single-cell chromatin accessibility sequencing (scATAC-seq)

Chromatin accessibility is related to the transcriptional activity and regulation of genes. scATAC-seq has gradually become one of the most widely used open region sequencing techniques due to its simple operation and high sensitivity. Xu et al. described a scATAC-seq technique based on flow cell sorting and 384-well plates which was characterized by its simplicity, reliability, and low cost (53). In addition, Tang Fuchou’s research group were the first to report single-cell chromatin accessibility sequencing technology based on the third-generation sequencing platform which they referred to as scNanoATAC-seq; this technique was able to achieve the simultaneous detection of chromatin open status and genomic structural variation in a single cell (54). Based on the development of this technology, researchers used scATAC-seq sequencing technology to analyze more than 600,000 human cells from 30 adult tissue types, and mapped a single-cell chromatin accessibility map for the human genome (55). Other researchers constructed a map of the renal single-cell chromatin landscape by applying scATAC sequencing (56). Single-cell chromatin accessibility sequencing technology will continue to evolve in the future and play an important role in both life science research and clinical medicine. With the further improvement of this technology and the promotion of this application, it will be possible to gain a deeper understanding of the functionality and regulatory network of cells, thus providing new concepts for the prevention and treatment of diseases.

#### 2.3.3 Single-cell histone modification sequencing

Histone modification is a common epigenetic modification that plays a key role in the regulation of gene transcription and expression. Histone modification plays an important role in the regulation of cell type-specific gene expression in multicellular organisms. With the advancement of platform technologies such as 10X and BD, several technologies have been developed to determine histone modification at the single-cell level. For example, Bartosovic et al. developed and applied the single-cell Cut&Tag (scCUT&Tag) method based on the scATAC-seq platform of 10x Genomics to investigate the histone modification profile at the single-cell level in the mouse brain (57). Another research team proposed that scChIX-seq could be used to map multiple histone markers in a single cell (58).

#### 2.3.4 Single cell Hi-C

This is a technique used to investigate the three-dimensional structure of chromosomes in a single cell. Hi-C is a high-throughput method to determine the spatial structure of chromosomes. Experimental techniques are advancing rapidly alongside developments in computing capability. The analysis of Hi-C data has represented a significant challenge. In 2022, a team developed a new algorithm called Higashi (hypergraph representation learning), a form of machine learning that can recognize three-dimensional (3D) objects 3D (59). Other researchers have developed a high-throughput imaging method for investigating single-cell chromatin epigenetic modification; this technique was referred to as Epigenomic MERFISH and was able to achieve high spatial resolution and high genomic resolution, and detect the epigenome *in situ* in single cells (60).

### 2.4 Single-cell multimodal omics

The rapid advancement and reduced cost of sequencing technology have made it possible to integrate several sequencing technologies. Single-cell multi-omics refers to simultaneously measuring multiple omics features at the single-cell level, including gene expression, DNA methylation, chromatin structure, and protein expression. This strategy could provide a more comprehensive, accurate, and detailed analysis of cells, thereby enhancing our understanding of cellular function and regulatory mechanisms. Multi-omics technology and single-cell sequencing technology are interdependent. Single-cell sequencing provides a data foundation for multi-omics technology, while single-cell multi-omics technology provides a more comprehensive cell analysis procedure by simultaneously measuring multiple omics features.

In 2017, Tang Fu Chou’s team developed a single-cell multi-omics technology (scCOOL-seq) that was able to simultaneously analyze chromatin state/nucleosome positioning, DNA methylation, genomic copy number variation, and ploidy in a single cell (61). This was the first analysis of up to five levels of genomic and epigenomic features within the same single cell. Other studies have mapped the epigenetic and transcriptional profile of immunity against a human influenza vaccine at the single-cell level by applying scATAC-seq and scRNA-seq analyses (62). With the development of cutting-edge technologies such as single-cell technologies, CUT&tag has also been applied to the single-cell level. For example, Professor Fan Rong’s team combined CUT&Tag technology and spatial omics to conduct high-spatial resolution analysis of epigenomics in mouse embryos at the E11 stage (63). In addition, other research groups have also developed a variety of single-cell multi-omics technologies, including the Paired Tag system developed by Ren Bing’s research group, which can jointly sequence single-cell gene expression and histone modification in order to study cell-specific chromatin status and gene expression regulatory mechanisms from complex tissue samples (64). In a recent study, Liu et al. reported a novel single-cell multi-omics technique known as HiRES; this was the first sequencing method to achieve the simultaneous detection of the transcriptome and three-dimensional genome at the single-cell level (65).

In summary, it is highly evident that single-cell multi-omics technology has become a rapidly developing frontier field over recent years, and some important progress has been achieved. There is still a lot of room for single-cell sequencing to be developed in the future. In addition to the gradual reduction of sequencing costs, further development will lead to further improvements in the sensitivity, resolution and throughput of this technology; this will allow for the analysis and sequencing of a greater number of cells. Furthermore, an increasing number of analytical methods and algorithms will be developed to allow deeper the acquisition of deeper single-cell sequencing data, thus allowing the extraction of a greater body of information. In addition, future strategies should aim to integrate multi-omics data with the application of single-cell sequencing technology in a wider range of investigative fields.

## 3 The application of single-cell sequencing technology to analyse the heterogeneity of leukemia

Cell heterogeneity is one of the key characteristics of leukemia. Traditional homogeneous sample sequencing technology often fails to reveal the subtle differences between different cell subsets, while single-cell sequencing technology can analyze single cells to reveal inter-cell heterogeneity. This could allow us to understand the existence of different subsets of leukemia cells and their role in disease development. This will enhance our understanding of the development and progression of leukemia more comprehensively.

**Figure 4:**
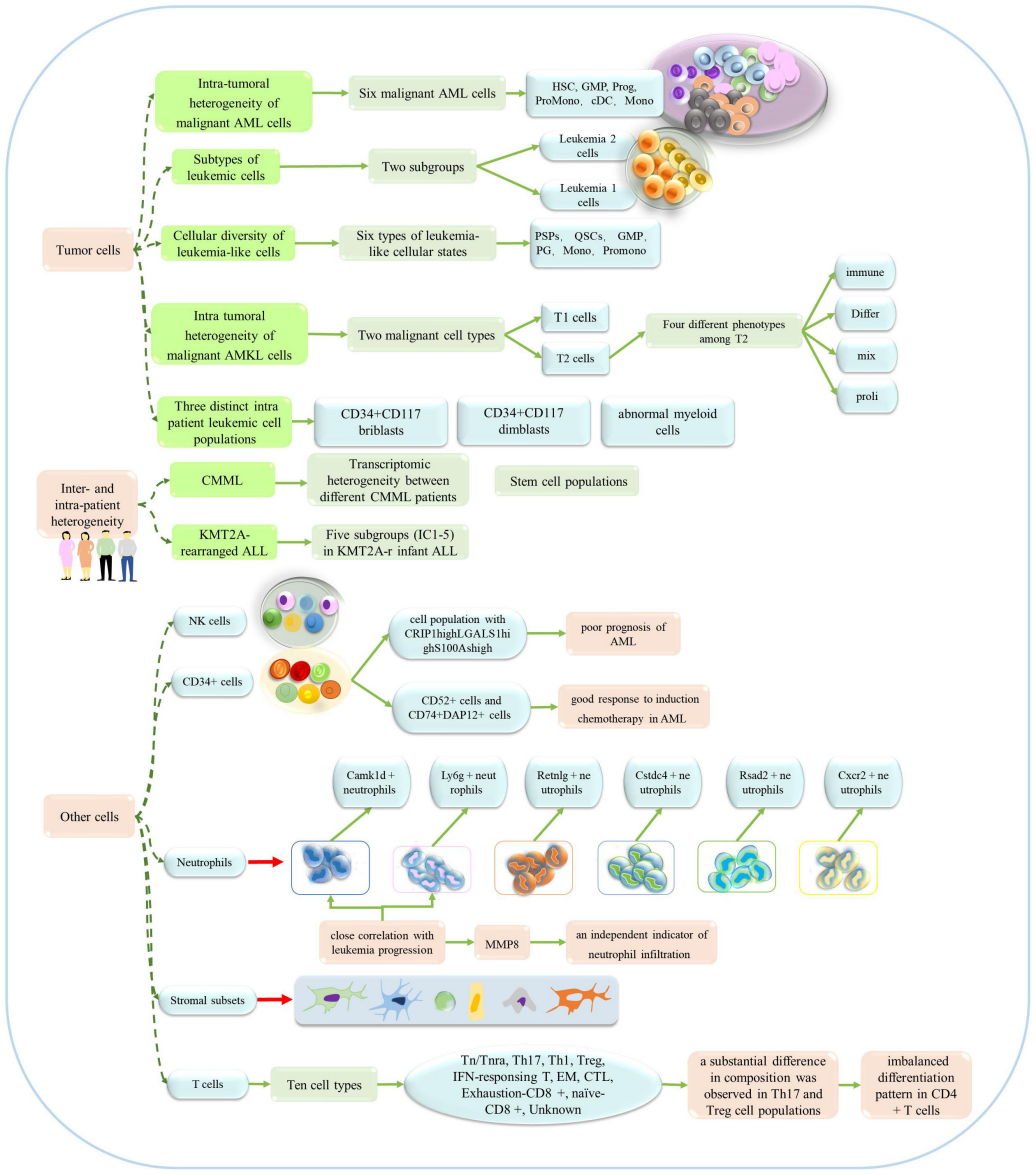
The application of single-cell sequencing in the heterogeneity of leukemia. This technology could reveal the heterogeneity of patients, tumors and other cells.

Over recent years, single-cell sequencing technology has been widely used to investigate the heterogeneity of leukemia. Single-cell sequencing technology can reveal intra-patient and inter-patient heterogeneity while defining intra-tumor heterogeneity, and providing insight into the detailed classification of other cell types (as shown in **Figure 4**). Previous researchers described a single-cell miRNA analysis technique that can be used to analyze AML heterogeneity with multiple phenotypes (66). Six malignant cell types (Hematopoietic stem cell (HSC)-like, Progenitor(Prog)-like, Granulocyte-macrophage progenitor(GMP)-like, promonocyte(Promomo)-like, Monocyte(Mono)-like or Conventional dendritic cell (cDC)-like malignant cells)were identified by scRNA-seq sequencing technology and genotyping studies, and their abundance varied widely across different AML genotypes (67). In a previous study, Thakral et al. investigated the nature and extent of transcriptional heterogeneity in pediatric patients with AML by applying single-cell sequencing and identified unique leukemia stem cell (LSC) clusters in each patient, thus revealing significant heterogeneity within each patient (68). Several other studies have demonstrated notable heterogeneity in patients with other types of leukemia, including chronic myelomonocytic leukemia, KMT2A-rearranged infant acute lymphoblastic leukemia (ALL), and t (8;21) AML (Jiang L et al. identified three distinct intrapatient leukemic cell populations that were arrested at different stages of myeloid differentiation: CD34+CD117dimblasts, CD34+CD117briblasts, and abnormal myeloid cells with partial maturation) (69–71).

Several studies have focused on the heterogeneity of leukemia tumor cells. For example, Saadatpour et al. used MLL-AF9-driven acute myeloid leukemia mice as a model and systematically characterized the heterogeneity within leukemia cells by single-cell analysis (The leukemic cells were further divided into two subgroups, Leukemia 1 cells, Leukemia 2 cells.) (72). Subsequent studies investigated multiple clinical samples and acquired a series of key results. To better understand intra-tumor heterogeneity in childhood acute lymphoblastic leukemia (cALL) patients, transcriptomic sequencing was used to investigate 39,375 individual cells from eight patients and three healthy children (73). Other researchers observed heterogeneity of cell status in bone marrow samples from patients with relapsed and refractory AML, and defined the intra-tumor heterogeneity of the transcriptome (Li K et al. identified six types of leukemia-like cellular states, including quiescent stem-like cells (QSCs), proliferating stem/progenitor-like cells (PSPs), granulocyte-monocyte progenitor (GMP), proliferating granulocyte (PG), promonocytes (promono), and differentiated monocytes (mono)) (74). Zhai et al. also obtained evidence to indicate the heterogeneity of tumors (75). It is worth noting that another study revealed the heterogeneity of pediatric acute megakaryoblastic leukemia (AMKL), but also identified potential markers of AMKL in children (Su N et al. termed the tumor clusters 3 and 5 as T1 and clusters 0, 1, 2, and 4 as T2, and they identified four major types of T2: “Differ” refers to differentiation, “immune” refers to immunity, “mix” refers to mix lineages, and “proli” refers to proliferation.) (76).In addition, regarding the heterogeneity of neutrophils, Hu T et al. revealed six different subpopulations of neutrophils, and in response to leukemia development, two neutrophil subpopulations showed a marked increase, which seems to be specifically related to the progression of leukemia (77).

In addition to investigating the heterogeneity of tumor cells, Baryawno et al. conducted a detailed study of bone marrow stromal cells and revealed the influence of AML on stromal cell population, and defined a total of six categories and 17 stromal cell subgroups (78). Leukemia stem cell/leukemia initiating cell (LIC) heterogeneity is a major obstacle to the treatment of AML. Two LICs-enriched cell subsets have been identified in Setd2-/–AML mice and the heterogeneity of LICs has been revealed (79). Other studies have investigated the heterogeneity of CML-SC (stem cells) and bone marrow CD34+ cells in AML patients, and NK cells from the bone marrow of AML patients and healthy individuals (80–82).

Many studies have investigated the heterogeneity of leukemia. However, most of these studies related directly to l tumors and tumor-specific stem cells. However, existing data also provide significant inspiration for future research; in addition to tumor cells, it is clear that we should investigate different stages of disease progression, changes in other cell types, and identify the specific molecular mechanisms involved; such research may allow us to develop more effective treatment strategies. Therefore, cell heterogeneity could play an important role in the development and treatment of leukemia. On the one hand, cellular heterogeneity may explain why some patients respond well to certain treatments while others are insensitive. For example, certain subtypes of leukemia cells may have specific mutations that render them sensitive to certain targeted therapeutic drugs. On the other hand, cell heterogeneity may be related to drug resistance or relapse in some patients during treatment, because different subpopulations of leukemia cells may have different mechanisms of resistance. In future studies, cell heterogeneity may provide key guidance for individualized therapy.

## 4 The application of single cell sequencing in immunotherapy for leukemia

Immunotherapy is a significant research hotspot in the treatment of blood tumors. Immunotherapy can inhibit the growth and spread of leukemia cells by regulating or enhancing the function of the patient’s own immune system. Immunotherapy has made significant progress in terms of the development of immune checkpoint inhibitors (ICB), CAR-T, novel leukemia vaccines, bi-specific antibodies and many other aspects (83, 84). Over recent years, single-cell sequencing technology has significantly enhanced our understanding of the immune microenvironment of leukemia patients, along with the molecular characteristics and functional status of their immune cells, but could also reveal the potential mechanisms of leukemia immunotherapy, thus providing important information relating to the monitoring and evaluation of immunotherapy, and is expected to provide important support for the precision treatment of leukemia.

Wu et al. performed scRNA-Seq on bone marrow cells from five NK-AML (M4/M5) patients and one normal donor, and identified eight T cell clusters (85). Another study retrieved scRNA-Seq data from 128,688 published cells (from 29 bone marrow donors, including 21 AML patients and eight healthy donors), comprehensively analyzed T/NK subsets, and re-clustered and annotated six major subsets. In addition, Hu et al. used scRNA-seq to analyze the PBMC microenvironment of five AML patients and six healthy donors receiving different chemotherapy regimens (86, 87). These data provided valuable insights into the immune microenvironment of leukemia; this information was crucial for the development of immunotherapy. A range of cell surface tumor antigens, such as CD33, CD123, CLL-1, and CD70, have been explored for the treatment of leukemia (88, 89). However, previous CAR-T cell therapies targeting these antigens have shown only limited clinical efficacy. In 2023, Gottschlich et al. identified two potentially effective CAR T target molecules for the treatment of AML (CSF1R and CD86) by comparing RNA sequencing data from over 500,000 single cells from 15 AML patients and nine healthy individuals (90). This study suggested that CAR-T cells precisely targeting these two proteins may have potential therapeutic significance for AML patients. Furthermore, by applying single-cell sequencing technology, Zhang et al. established a comprehensive cell atlas for one patient with Philadelphia chromosome-like acute lymphoblastic leukemia (Ph-like ALL) with a novel TPR-PDGFRB fusion gene at both diagnosis and relapse; this study provided useful information relating to the future development of immunotherapeutic techniques (91). In addition, Gao et al. applied single-cell TCR sequencing (scTCR seq) and scRNA seq to investigate patients with T-cell large granular lymphocyte leukemia (T-LGLL) and revealed abnormal cell survival and apoptosis gene programs in T-LGLL (92). Other studies discovered that T cells in B-cell acute lymphoblastic leukemia (B-ALL) showed more immune pathway activation features when compared to healthy individuals, and identified six CD8+T clusters, six NK clusters, and six Mac clusters that were all related to aggrephagy (93, 94). These data contribute to a better understanding of the potential mechanisms of targeted immunotherapy for leukemia, and also revealed the importance of aggregation-related patterns in the tumor microenvironment with regards to immunotherapy.

In summary, the application and significance of immunotherapy in leukemia lies in providing patients with a new treatment option that can effectively enhance the patient’s own immune system to combat leukemia cells, thereby improving treatment outcomes and increasing survival and quality-of-life. Compared to traditional chemotherapy and radiotherapy, immunotherapy generally has fewer toxic side effects and can reduce the recurrence and metastasis of leukemia, at least to a certain extent. The advancement of single-cell sequencing technology also plays a role in promoting the development of leukemia immunotherapy.

## 5 Application of single cell sequencing in the drug resistance and chemotherapy of leukemia

The resistance of leukemia cells to therapeutic drugs may lead to poor treatment outcomes or relapse. The resistance of leukemia cells may be associated with a range of factors, including genetic mutations, epigenetic changes, and abnormal cell signaling pathways. Single-cell sequencing technology plays an important role in studying the resistance of leukemia to certain drugs. By performing single-cell sequencing, it is possible to perform high-resolution analysis of leukemia cells; this strategy may identify heterogeneity between different subgroups of cells, thus revealing the characteristics and mechanisms of drug-resistant cell populations. This will help to enhance our understanding of the process that leads to drug resistance and will provide a theoretical basis for the development of new treatment strategies.

**Figure 5:**
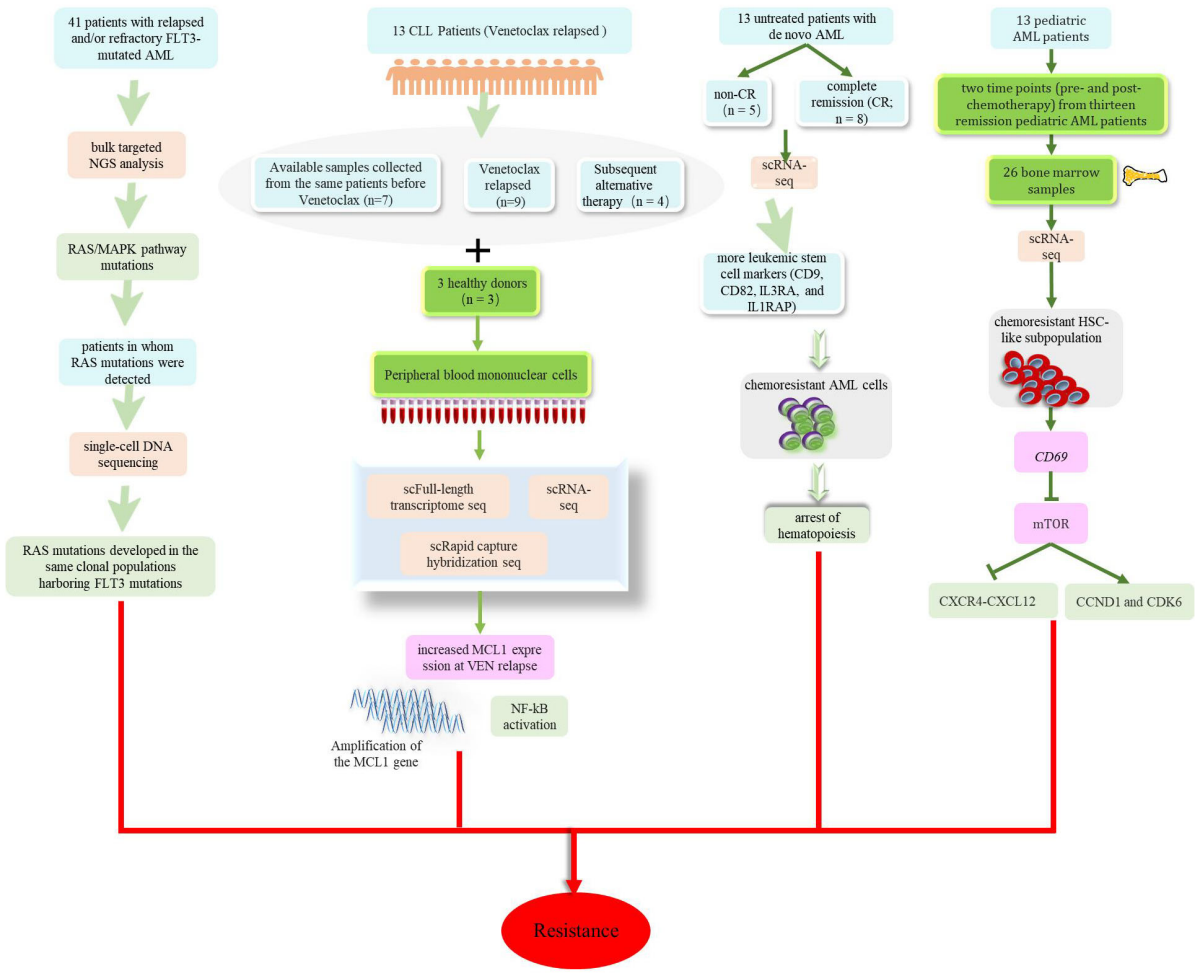
Potential mechanisms of drug resistance in patients with leukemia including mutations related to the Ras/MAPK signaling pathway occurring in patients after treatment with Gilteritinib. Increased MCL1 expression at VEN relapse was only partially explained by amplification of the MCL1 gene and increased MCL1 expression driven by NF-kB. AML cells that were resistant to chemotherapy showed premature accumulation during the early hematopoietic process. CD69 mediates drug resistance by regulating the mTOR-CCND1-CXCR4 axis.

Research has identified a drug-resistant HSC-like stem cell subpopulation characterized by the CD69 surface marker in patients with leukemia, and has preliminarily revealed that CD69 mediates drug resistance by regulating the mTOR-CCND1-CXCR4 axis (95). In order to identify potential resistance mechanisms to “7 + 3” induction chemotherapy in AML, Cheng et al. utilized scRNA-seq methods and revealed that hematopoietic obstruction occurs earlier in drug-resistant AML cells (96). Various drugs are used to treat leukemia, including Venetoclax, tyrosine kinase inhibitors (TKIs), Gilteritinib, and Quizartinib; all of these drugs can lead to certain clinical drug resistance. Multiple studies have revealed changes in tumor cell clones of patients after drug treatment and have allowed investigation of the complexity of drug resistance and different response mechanisms (97–101). As shown in **Figure 5**, these findings enhanced our understanding of resistance to leukemia drugs and provided new treatment strategies to overcome treatment resistance and relapse in leukemia. Furthermore, Li et al. discovered that LGALS1 is a promising target for the resistance of AML to chemotherapy. The administration of LGALS1 inhibitors to primary AML cells derived from patients, cell lines, and AML animal transplant models could help to eliminate QSCs (a cell population involved in resistance to AML chemotherapy and poor prognosis) and enhance the efficacy of chemotherapy (74). However, it is worth noting that, despite researchers validating the role of LGALS1 in AML chemotherapy resistance through various experimental methods, further clinical research is still needed to evaluate the feasibility and effectiveness of LGALS1 as a potential treatment target. The use of single-cell sequencing technology could provide a deeper understanding of the molecular mechanisms responsible for leukemia drug resistance and allow the discovery of new therapeutic targets.

## 6 Application of single cell sequencing in the discovery of potential therapeutic targets and markers for leukemia

By identifying specific markers and targets of leukemia cells, researchers could develop targeted therapeutic drugs for these molecules, thus achieving more individualized and precise treatment options. Traditional genomics research typically analyzes cell populations as a whole, while single-cell sequencing technology focusses at the level of individual cells, thereby facilitating the discovery of more precise treatment targets and markers.

On the basis of constructing a mouse model that overexpressed RalA GTPase, Professor Fei Jia’s team used single-cell transcriptomic technology to detect the effect of RalA overexpression on the self-renewal of bone marrow hematopoietic stem/progenitor cells in normal mice, and provided a theoretical basis for the clinical use of RalA inhibitors in the treatment of CML (102). Zhang et al. were the first to use single-cell sequencing technology to map the single-cell atlas of minimal residual disease (MRD) in pediatric B-ALL and validated the hypoxia signaling pathway as a potential therapeutic target for B-ALL treatment by investigating both *in vivo* and *in vitro* experimental models (103). As shown in **Figure 2**, while previous studies have confirmed the existence of numerous therapeutic targets for leukemia, research on prognostic markers for this disease are relatively limited. By applying single-cell sequencing, Crinier et al. identified CD160 as an important marker that was significantly associated with AML prognosis (82). Furthermore, by applying scRNA-seq, Xiong et al. identified several potential biomarkers for the clinic: AT-rich interaction domain 2, lysine methyltransferase 2A, and synaptotagmin binding cytoplasmic RNA interacting protein; all of these could be used to predict the prognosis of t (8;21) AML (104). Collectively, these findings provide data to support the treatment and prognosis of this disease, while single-cell sequencing technology provides more comprehensive and in-depth information for leukemia research at the cellular level; this provides important technical support for targeted therapy and precision medicine.

## 7 The application of single-cell sequencing technology in the clonal evolution of leukemia tumors

Leukemia tumors arise mostly due to genetic mutations; thus, tumor cells will constantly divide and proliferate, forming multiple clones. These clones may evolve during treatment, leading to increased tumor heterogeneity. The clonal evolution of leukemia tumors is a complex process, including initial cloning, subclonal expansion and other stages; these stages need to be investigated and monitored by a variety of methods to guide the formulation and adjustment of treatment programs. Over recent years, and alongside the progress of molecular biology and genetics, the development of single-cell sequencing technology has provided a new breakthrough for the investigation of clonal evolution in leukemia tumors. By performing single-cell sequencing technology, it is possible to explore genomic variations and differences in expression between different subclones within the population of cells in leukemia tumors, thus revealing the evolutionary patterns of tumor clones.

Research on the progression of AML used to focus predominantly on genetic changes at the DNA mutation level, primarily in a large number of AML cells, thus demonstrating the existence of DNA clonal evolution. Stetson et al. utilized scRNA-seq to assess RNA changes in LIC from matched patient diagnosis and relapse samples, thus revealing the phenomenon of RNA clonal evolution in LIC during the progression of AML (105). In addition, a research team led by Takahashi used Mission Bio’s Tapestri single-cell sequencing platform, combined with traditional bulk sequencing, to analyze 154 bone marrow mononuclear cell samples from 123 patients with AML, and found that approximately half of the mutations in AML patients exhibited linear clonal evolution (a classic cancer development model), while the other half exhibited branched clonal evolution (106). AML patients not only exhibit the phenomenon of clonal evolution in their bodies but also exhibit different evolutionary pathways. Research has also revealed clonal heterogeneity and clonal evolution in other forms of leukemia (107–109). For example, researchers at MD Anderson Cancer Center used the single-cell analysis platform to conduct disease research on AML patients at different stages (initial diagnosis, complete recovery, and relapse) and revealed different clonal evolution processes in different patients (45). Comprehensive analysis of the early detection and characterization of newly expanded subclones may help to deepen our understanding of the development of this disease. Notably, Hughes et al. sequenced individual cells from three MDS-derived AML patients; this research helped to enhance our understanding of genetic changes during MDS to AML transition and in the development of targeted therapies for initiating and dominant clones (110). The application of single-cell sequencing technology in the clonal evolution of leukemia tumors holds certain clinical value, with important implications for guiding treatment, predicting tumor evolution trends, and enhancing our understanding of tumor biology.

## 8 Other applications

Single-cell sequencing technology has been extensively studied in leukemia. In addition to the application of cell heterogeneity, immunotherapy, and drug-resistant chemotherapy mentioned in this review, other have focused on the in-depth exploration of the pathogenesis of leukemia, the identification of new cell populations, and early response evaluation.

First, the use of single-cell sequencing could reveal the mechanisms responsible for disease occurrence. For example, Baryawno et al. investigated the impact of AML on stromal cells and revealed that cancer cells regulate specific stromal cells to impair normal tissue functionality, thus promoting carcinogenesis (78). Furthermore, researchers have identified mutations in LCP1 and WNK1 as new drivers of CLL and demonstrated their impact on the CLL pathway (111). In addition, scRNA-seq could identify new cell populations; Diemer et al. identified a new cell population in the pre-leukemic state by demonstrating the expression of genes related to immune activation (112). NK cell activity has often been shown to be impaired in AML patients; recent studies have demonstrated that NK-cell dysfunction may be related to the renin-angiotensin system (RAS) and that neurotransmitters seem to be crucial for the development of AML (113). A previous study analyzed scRNA-seq data from 16,843 bone marrow mononuclear cells (BMMCs) from healthy individuals and AML patients and revealed the widespread involvement of alternative polyadenylation (APA) regulation in the development of leukemia and erythropoiesis (114). scRNA-seq analysis also confirmed the indispensable role of the mature and terminal, CD56 bright, and transitional subsets of NK cells in maintaining treatment-free remission in CML patients (115). Finally, a mass cytometry study of 32 AML patients revealed that the reduction of extracellular-signal-regulated kinase (ERK) 1/2 and p38 mitogen-activated protein kinase (MAPK) phosphorylation in myeloid cells 24 hours after chemotherapy was an important predictor of 5-year overall survival (116). Therefore, it is evident that the development of single-cell sequencing technology provides us with significantly more possibilities and hope for the treatment of leukemia.

## 9 Conclusion

The advances and applications of single-cell sequencing technology provide powerful tools for studying the heterogeneity, classification, molecular mechanisms, personalized treatment, and monitoring of leukemia. These applications not only deepen our understanding of leukemia subtypes but also open up new possibilities for future clinical applications. However, there are also some drawbacks and challenges to the use of this technology. First, the complexity of data analysis. Single-cell sequencing can generate a large amount of data, thus requiring complex data analysis and bioinformatics processing. Second, single-cell sequencing technology is relatively costly when compared to traditional bulk sequencing techniques, including both equipment and experimental consumables. These costs may limit the widespread application of this technology in clinical practice. Third, it is sometimes difficult to acquire appropriate high-quality samples. In certain types of leukemia, the limited number of leukemia cells may restrict sample acquisition, thus affecting the application of single-cell sequencing technology. Fourth, technical standardization and stability can represent problems. The standardization and stability of single-cell sequencing technology across different laboratories and operators remains a significant challenge that could potentially influence the reproducibility and comparability of results. Finally, the bioinformatics analysis of single-cell sequencing data requires users to handle a large amount of data, necessitating specialized bioinformatics analysis skills. Future research directions may focus on continuous improvement and refinement in technology, cost, data analysis, and emphasize multi-omics analysis, the establishment of spatial distribution maps, and the development of personalized treatment strategies. In conclusion, with the continuous development and improvement of technology, it is believed that single-cell sequencing technology will play an increasingly important role in the research of not only leukemia but the treatment of several diseases.
